# Supplementary material for: Structural basis of QueC-family protein function in qatABCD anti-phage defense
Source: Nat Commun. 2026 Apr 20;17:5420. doi: 10.1038/s41467-026-72155-8 (PMC13279940; doi:10.1038/s41467-026-72155-8)
Supplement: Supplementary file 1 — Supplementary Information [file 41467_2026_72155_MOESM1_ESM.pdf]

Supplementary Information for

Structural basis of QueC-family protein function in qatABCD anti-phage defense

Angela Gao[1,2,†], Douglas R. Wassarman[1,2,†], Philip J. Kranzusch[1,2,3*]

Corresponding author: philip_kranzusch@dfci.harvard.edu

This PDF file includes:

Supplementary Figures 1–8
Supplementary Tables 1–3

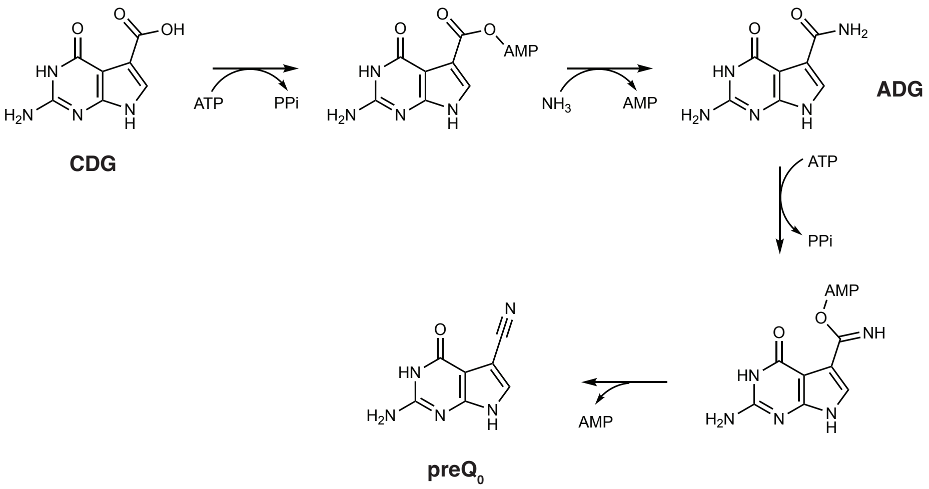

Supplementary Figure 1 | Canonical QueC reaction mechanism
QueC catalyzes activation of 7-carboxy-7-deazaguanine (CDG) using one molecule of ATP. Nucleophilic attack by ammonia displaces AMP to generate the intermediate product 7-amido-7-deazaguanine (ADG). Another molecule of ATP is consumed to generate the final QueC product 7-cyano-7-deazaguanine (preQ_0).

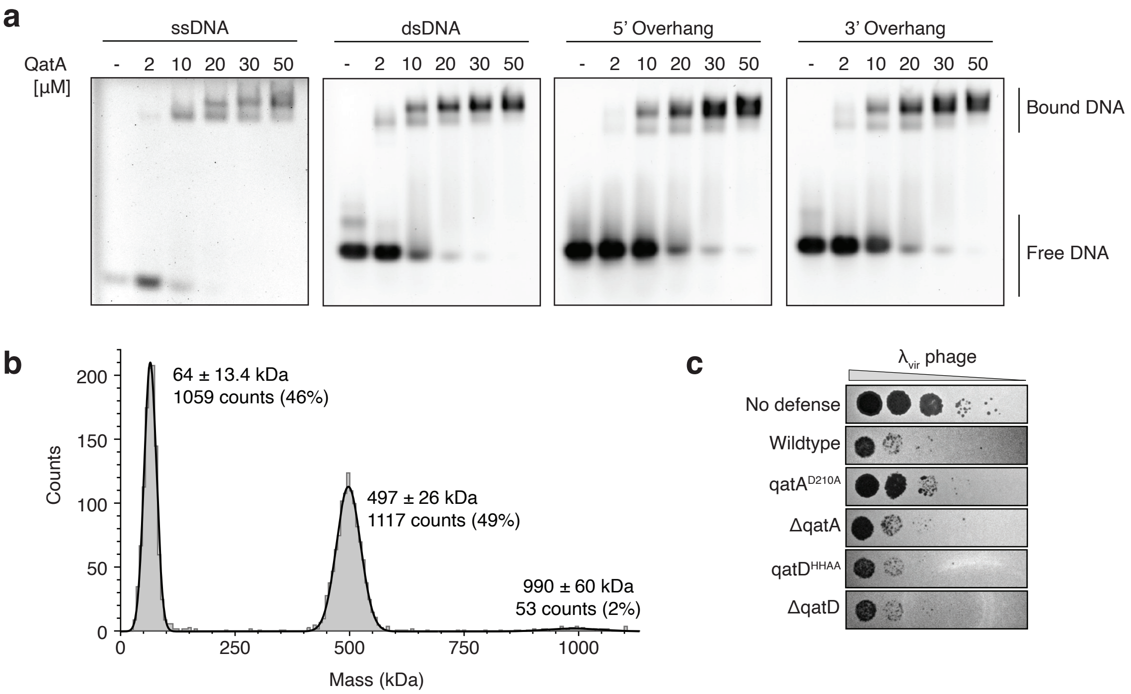

Supplementary Figure 2 | QatA and QatD in qatABCD-mediated anti-phage defense
a, Electrophoretic mobility shift assays of QatA with ssDNA, dsDNA, 5′ overhang, and 3′ overhang substrates run on 2% TB-agarose gels stained with ethidium bromide. Protein concentrations are expressed as µM of QatA monomer. **b,** Mass photometry of His-tagged QatA expressed in the full qatABCD operon. Calculated molecular weight of QatA monomer is 72.9 kDa, heptamer is 510.3 kDa, and octamer is 583.2 kDa. Raw data are provided as a Source Data file **c,** Representative plaque assays of *E. coli* expressing GFP control (no defense) or *P. aeruginosa* qatABCD with the indicated genotypes (n=5). QatD H15A/H17A (qatD^{HHAA}).

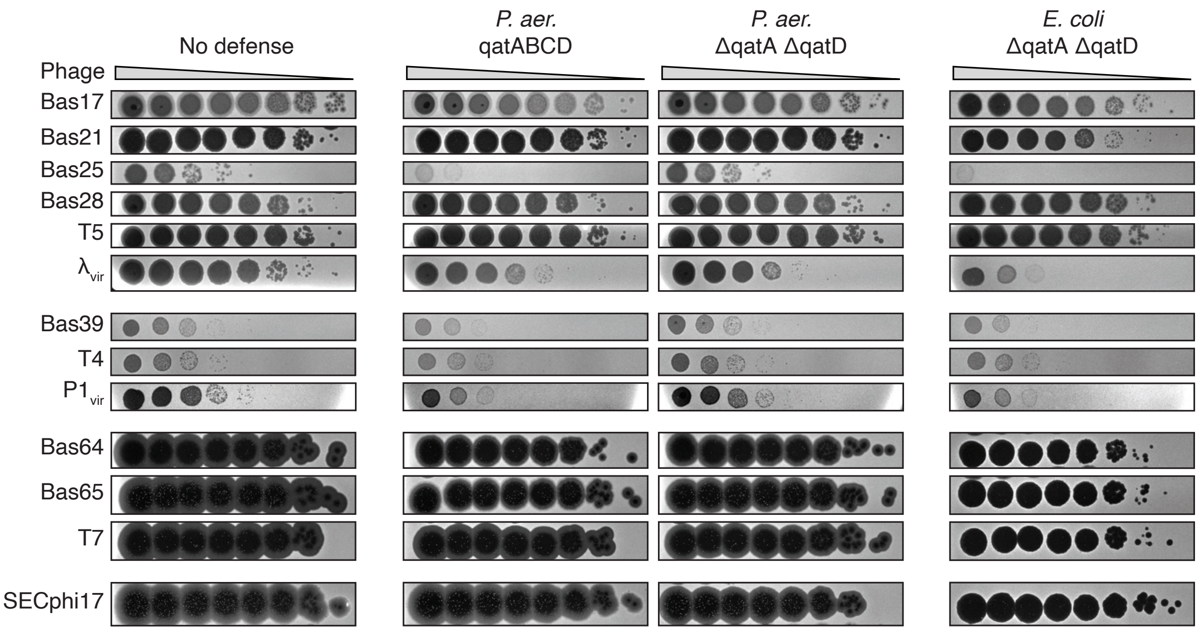

Supplementary Figure 3 | QatBC anti-phage defense panel

Representative plaque assays of *E. coli* expressing a GFP control (no defense), *P. aeruginosa* qatABCD operon, *P. aeruginosa* qatBC alone, or *E. coli* qatBC alone (n=3). Double-stranded DNA phages are grouped by morphology (siphoviruses, myoviruses, podoviruses) and listed separately from the single-stranded DNA phage SECphi17.

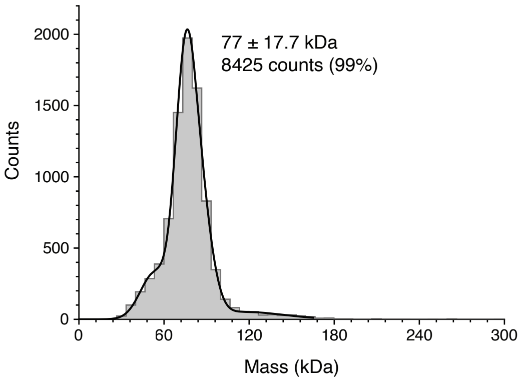

Supplementary Figure 4 | QatB and QatC form a 1:1 complex
Mass photometry of the QatBC complex. Calculated molecular weight of QatB is 29.7 kDa and His-tagged QatC is 51.6 kDa. Raw data are provided as a Source Data file.

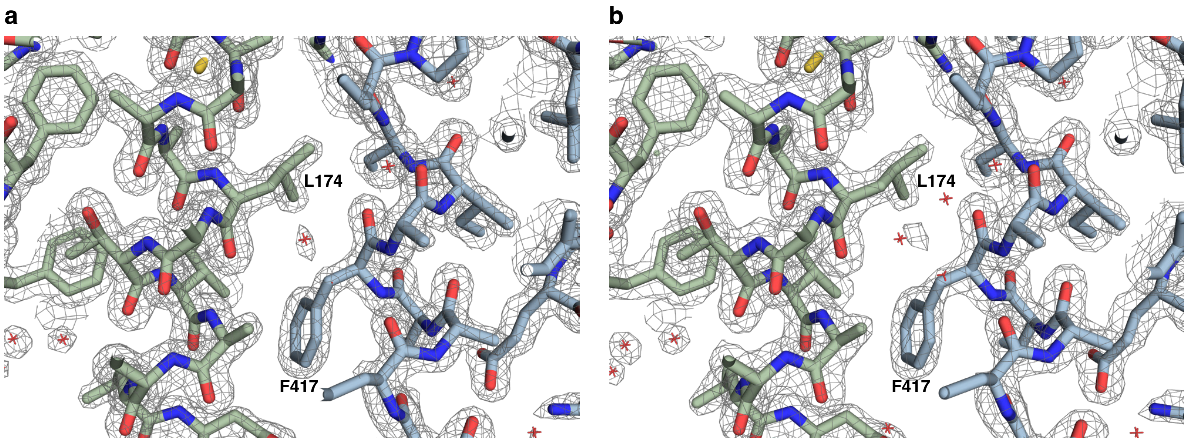

Supplementary Figure 5 | Crystal structure electron density

Representative electron density maps shown at the QatB–QatC interface for **a,** apo and **b,** ATP-bound crystal structures. QatB is shown in green and QatC is shown in blue. $2F_O{-}F_C$ electron density mesh is displayed at σ=1.5.

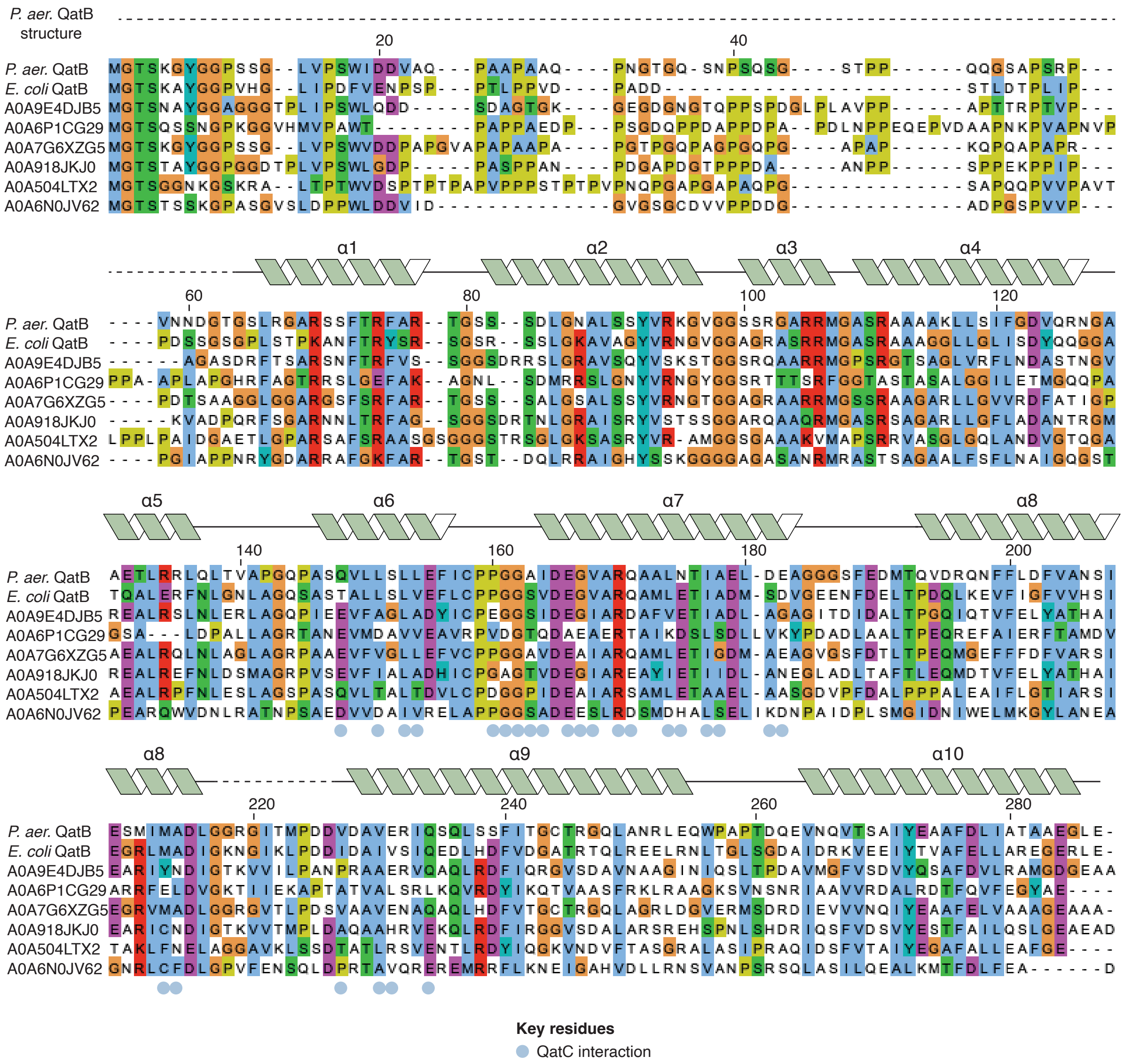

Supplementary Figure 6 | QatB sequence alignment

Sequence alignment of representative QatB sequences (UniProtKB accession numbers shown). Secondary structure is annotated from *P. aeruginosa* QatB structure in complex with QatC. Alignment is colored using ClustalX color scheme based on side chain properties. Residue positions are numbered according to the *P. aeruginosa* sequence. QatB residues found within 4.0 Å of QatC in the QatBC apo structure are indicated with blue dots.

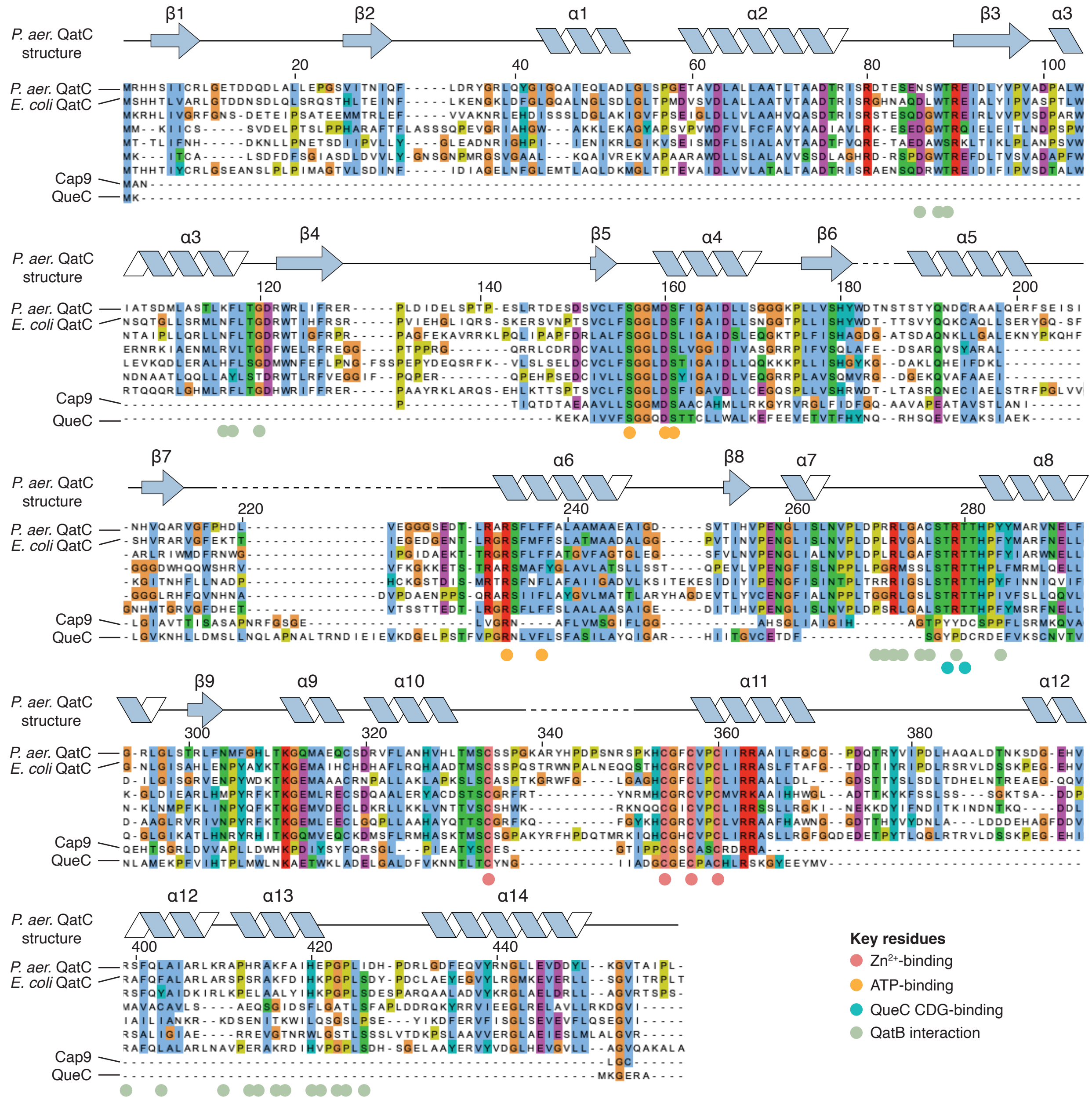

Supplementary Figure 7 | QatC sequence alignment

Sequence alignment of representative QatC sequences (UniProtKB accession numbers: A0A2P6WBR3, A0A1E2V6V1, A0AA91FJV8, A0A158EPR1, A0A7X2LQZ7), *Rhizobiales sp.* Cap9, and *B. subtilis* QueC. Secondary structure is annotated from *P. aeruginosa* QatC structure in complex with QatB. Alignment is colored using ClustalX color scheme based on side chain properties. Residue positions are numbered according to the *P. aeruginosa* sequence. Cysteine residues involved in Zn^{2+}-binding are indicated with red dots, residues involved in ATP-binding are indicated with orange dots, residues involved in QueC CDG-binding are indicated with teal dots, and residues found with 4.0 Å of QatB in the QatBC apo structure are indicated with green dots.

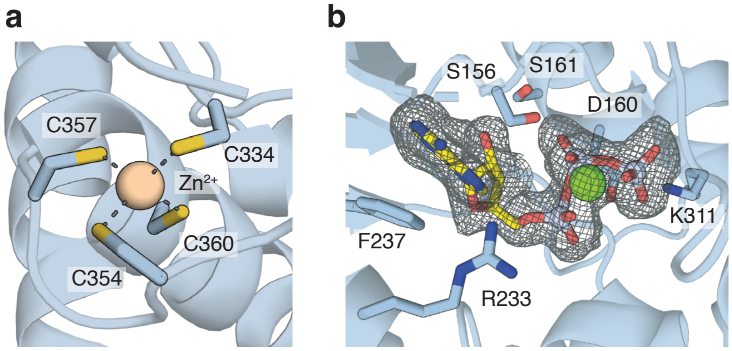

Supplementary Figure 8 | QatC ligand sites

a, Conserved cysteine residues C334, C354, C357, and C360 coordinate Zn^{2+} in the conserved QueC domain of QatC in the QatBC complex. **b,** Gray mesh depicts F_O−F_C polder omit map of ATP and Mg^{2+} omitted from QatBC–ATP structure (σ=3).

Supplementary Table 1 | QueC protein accessions

Operon type	Organism	QueC accession number	Reference database
qatABCD	*Pseudomonas aeruginosa*	WP_003160498.1	RefSeq
qatABCD	*Escherichia coli*	WP_038416547.1	RefSeq
type IV CBASS	*Rhizobiales sp.*	2624355116	IMG
type IV CBASS	*Bacillus subtilis*	2690214061	IMG
queCDEF	*Bacillus subtilis*	WP_003245417.1	RefSeq

Supplementary Table 2 | Crystallographic statistics

Structure	QatB-QatC apo	QatB-QatC ATP
PDB accession	9ZEF	9ZEE
Wavelength (Å)	0.9199	0.9792
Resolution range (Å)	66.81 - 1.55 (1.58 - 1.55)	67.15 - 1.81 (1.84 - 1.81)
Space group	$P2_12_12_1$	$P2_12_12_1$
Unit cell (Å)	70.892 74.61 133.629	70.704 74.366 134.304
Total reflections	889266 (42129)	799614 (38314)
Unique reflections	102996 (5114)	64929 (3179)
Multiplicity	8.6 (8.2)	12.3 (12.1)
Completeness (%)	100.0 (100.0)	99.8 (99.5)
Mean I/sigma(I)	12.3 (1.6)	9.0 (1.7)
Wilson B-factor (Å²)	21.68	25.89
R-merge	0.073 (1.071)	0.151 (1.275)
R-meas	0.077 (1.144)	0.157 (1.331)
R-pim	0.026 (0.399)	0.044 (0.379)
CC1/2	0.999 (0.767)	0.998 (0.778)
CC*	1 (0.93)	0.999 (0.932)
Reflections used in refinement	102989 (3371)	64925 (2651)
Reflections used for R-free	5280 (182)	3300 (127)
R-work	0.1767 (0.2766)	0.1812 (0.2412)
R-free	0.1930 (0.2873)	0.2035 (0.2868)
Number of non-hydrogen atoms	5542	5488
macromolecules	4949	4885
ligands	1	33
solvent	592	570
Protein residues	646	638
RMS (bonds) (Å)	0.011	0.006
RMS (angles) (degrees)	1.1	0.77
Ramachandran favored (%)	97.95	97.92
Ramachandran allowed (%)	2.05	2.08
Ramachandran outliers (%)	0	0
Rotamer outliers (%)	0.38	0.39
Clashscore	3.06	3.18
Average B-factor (Å²)	31.61	34.07
macromolecules	30.63	33.19
ligands	26.92	31.63
solvent	39.82	41.76

Supplementary Table 3 | Plasmids and primers used in this study

Plasmid name	Construction method	Forward primer	Reverse primer	Template	Description
pAG001_pBAD02_sfGFP	Synthesis (Twist Biosciences)				sfGFP
pAG003_pBAD02_EcoliNCTC9009_qatABCD	Synthesis (Twist Biosciences)				*E. coli* qatABCD
pAG004_pBAD02_PaerK6156_qatABCD	Synthesis (Twist Biosciences)				*P. aeruginosa* qatABCD
pAG006_pBAD02_qatABCD_SSAA	Site-directed mutagenesis	gggtgggatggacgctttcattggggcgatcgatctactc	gtccatcccacccgcgaacaggcagacgctatcgg	pAG004_pBAD02_PaerK6156_qatABCD	QatC S156A/S161A
pAG007_pBAD02_qatACD	Site-directed mutagenesis	ggattgatatgaggcaccacagcattatctg	gtgcctcatatcaatccttatgatttcccgctgaattc	pAG004_pBAD02_PaerK6156_qatABCD	ΔqatB
pAG008_pBAD02_qatABCD_G2D	Site-directed mutagenesis	gatatggatacctccaaaggatatggaggcc	tttggaggtatccatatcaatccttatgatttcccgctg	pAG004_pBAD02_PaerK6156_qatABCD	QatB G2D
pAG011_pETC6_qatBC	Gibson	Insert: GTTTAACTTTAAGAAGGAGATATACATatgggtacctccaaaggatatggag, Vector: GGATCCCACCATCACCATCACC	Insert: TCAATGGTGATGGTGATGGTGGGATCCtagaggtatagctgtgacacccttg, Vector: ATGTATATCTCCTTCTTAAAGTTAAACAAAAGAGCTC	Insert: pAG004_pBAD02_PaerK6156_qatABCD, Vector: pETC6	QatBC-His purification
pAG012_pBAD02_qatAHisBCD	Site-directed mutagenesis	CACCATCACCATtaaCAGGAGGAATTAACCatgggtacctccaaaggatatggag	CTGttaATGGTGATGGTGATGGTGGGATCCtgatttcccgctgaattcaagtacg	pAG004_pBAD02_PaerK6156_qatABCD	QatA-His purification in operon; cloning intermediate
pAG015_pBAD02_qatABdel16-22CD	Site-directed mutagenesis	ttggtggcacaaccagctgctccggccg	ggttgtgccaccaagccggagctcgggcc	pAG004_pBAD02_PaerK6156_qatABCD	QatBΔ16-22
pAG016_pBAD02_qatABCdel2-144D	Site-directed mutagenesis	ctggaatgagaacagatgagtccgatagcgtctgcctg	catctgttctcattccagcccctccgctgcagtc	pAG004_pBAD02_PaerK6156_qatABCD	QatCΔ2-144
pAG017_pBAD02_qatABCdel378-458D	Site-directed mutagenesis	gatgaagccgcagaaaagcccgattccaaagtgg	ctgcggcttcatcgggtctgatcggggcc	pAG004_pBAD02_PaerK6156_qatABCD	QatCΔ378-458
pAG023_pETC6_qatA	Gibson	Insert: TTAACTTTAAGAAGGAGATATACATatgttcctaaatgaccaggaaacgg, Vector: GGATCCCACCATCACCATCACC	Insert: AATGGTGATGGTGATGGTGGGATCCtgatttcccgctgaattcaagtacg, Vector: ATGTATATCTCCTTCTTAAAGTTAAACAAAAGAGCTC	Insert: pAG004_pBAD02_PaerK6156_qatABCD, Vector: pETC6	QatA-His purification
pAG026_pET_qatAHisBCD	Gibson	Insert: TTAACTTTAAGAAGGAGATATACATatgttcctaaatgaccaggaaacgg, Vector: TAAGcggCcGCTGCTAAC	Insert: GGGCTTTGTTAGCAGCgGccgCTTActaacgggacactaaatcacgc, Vector: ATGTATATCTCCTTCTTAAAGTTAAACAAAAGAGCTC	Insert: pAG012_pBAD02_qatAHisBCD, Vector: pETC6	QatA-His purification in operon

pAG033_pBAD02_qatABdel34-40CD	Site-directed mutagenesis	caaccaaactctcagtccggctctacgcctcctc	gactgagagtttggttgggcggccggagcag	pAG004_pBAD02_PaerK6156_qatABCD	QatBΔ34-40
pAG040_pETC6_EcoliNCTC9009_qatBC	Gibson	Insert: TTTAAGAAGGAGATATACATatgggaacctcaaaagcttacgg, Vector: GAATTCCCAGGCATCAAATAAAACGAAAG	Insert: TGATGGTGATGGTGGGATCCtgtaaggggcctcgttatgactc, Vector: ATGTATATCTCCTTCTTAAAGTTAAACAAAAGAGCTC	Insert: pAG003_pBAD02_EcoliNCTC9009_qatABCD, Vector: pETC6	*E. coli* QatBC-His purification; cloning intermediate
pAG041_pBAD02_EcoliNCTC9009_qatBCHis	Gibson	Insert: GCTAACAGGAGGAATTAACCatgggaacctcaaaagcttacgg, Vector: GAATTCCCAGGCATCAAATAAAACGAAAG	Insert: TATTTGATGCCTGGGAATTCTTATCAATGGTGATGGTGATGGTGGGATC, Vector: GGTTAATTCCTCCTGTTAGCCC	Insert: pAG040_pETC6_EcoliNCTC9009_qatBC, Vector: pAG004_pBAD02_PaerK6156_qatABCD	*E. coli* QatBC-His purification; cloning intermediate
pAG048_pBAD02_qatABCD_qatAD210A	Site-directed mutagenesis	atcgttgctgaccttgatcgatgcctgcccaag	aaggtcagcaacgataactaccagttggtctatgtctgcg	pAG004_pBAD02_PaerK6156_qatABCD	QatA D210A
pAG049_pBAD02_qatABCD_qatDHHAA	Site-directed mutagenesis	gcctgcgctctcgacctttatcctgatcatgagcgcc	gagagcgcaggcgaaatccacccactttggaatcgg	pAG004_pBAD02_PaerK6156_qatABCD	QatD H15A/H17A
pAG050_pBAD02_qatBCD	Site-directed mutagenesis	AATTAACCatgggtacctccaaaggatatggaggcccg	aggtacccatGGTTAATTCCTCCTGTTAGCCCAAAAAACGG	pAG004_pBAD02_PaerK6156_qatABCD	ΔqatA
pAG051_pBAD02_qatABC	Site-directed mutagenesis	acctctatgaGAATTCCCAGGCATCAAATAAAACGAAAGGCTCAG	GGGAATTCtcatagaggtatagctgtgacacccttgagatagtc	pAG004_pBAD02_PaerK6156_qatABCD	ΔqatD
pAG068_pBAD02_qatBC	Site-directed mutagenesis	acctctatgaGAATTCCCAGGCATCAAATAAAACGAAAGGCTCAG	GGGAATTCtcatagaggtatagctgtgacacccttgagatagtc	pAG050_pBAD02_qatBCD	ΔqatA ΔqatD
pAG069_pBAD02_EcoliNCTC9009_qatBC	Site-directed mutagenesis	cgaggccccttacaTGAGAATTCCCAGGCATCAAATAAAACG	tgtaaggggcctcgttatgactccactcagcaggcg	pAG041_pBAD02_EcoliNCTC9009_qatBCHis	*E. coli* ΔqatA ΔqatD
pAG070_pBAD02_qatABCD_TTAA	Site-directed mutagenesis	cgcacgcgctacgcatccatactacatggcgcg	gtagcgcgtgcgctacatgcacccaggcgg	pAG004_pBAD02_PaerK6156_qatABCD	QatC T278YA/T280A
pAG071_pBAD02_qatABCD_TTYD	Site-directed mutagenesis	agctaccgcgatacgcatccatactacatggcgcg	gtatcgcggtagctacatgcacccaggcgg	pAG004_pBAD02_PaerK6156_qatABCD	QatC T278Y/T280D
pAG072_pBAD02_qatABCD_Cdel265-279	Site-directed mutagenesis	tctctgacgacgcatccatactacatggcgcg	gcgtcgtcagagagatcaggccattttcaggcacatg	pAG004_pBAD02_PaerK6156_qatABCD	QatC Δ265-279
pAG073_pBAD02_qatABCD_RRREEE	Site-directed mutagenesis	gaactcaaggaagcgcctcacgaggccaagttcgcaattcatgagc	ctcgtgaggcgcttccttgagttcagcgatggcgagctgg	pAG004_pBAD02_PaerK6156_qatABCD	QatC R407E/R410E/R414E
