# Supplementary material for: Structural basis of QueC-family protein function in qatABCD anti-phage defense
Source: Nat Commun. 2026 Apr 20;17:5420. doi: 10.1038/s41467-026-72155-8 (PMC13279940; doi:10.1038/s41467-026-72155-8)
Supplement: Supplementary file 2 — Description of Additional Supplementary Files [file 41467_2026_72155_MOESM2_ESM.pdf]

Description of Additional Supplementary Files

File name: Supplementary Data 1
Description: AlphaFold model. Trimmed AlphaFold 3 model used for molecular replacement.

File name: Supplementary Data 2
Description: AlphaFold model. Complete AlphaFold 3 model.
