# Supplementary figures and images for: Structural basis of QueC-family protein function in qatABCD anti-phage defense
Source: Nat Commun. 2026 Apr 20;17:5420. doi: 10.1038/s41467-026-72155-8 (PMC13279940; doi:10.1038/s41467-026-72155-8)

Supplementary Figure 2a. QatA EMSA

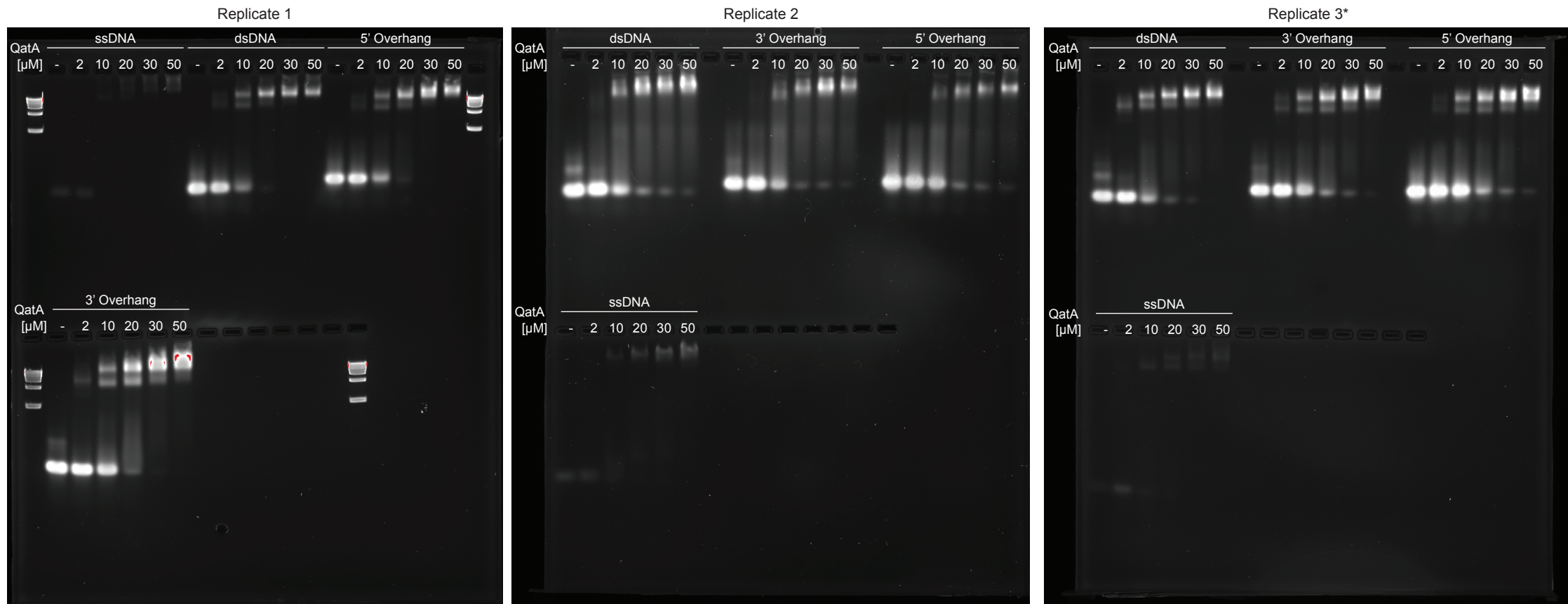

\*Shown in main figure

Supplement: Supplementary file 7 — Source Data [file 41467_2026_72155_MOESM7_ESM.zip › Source Data/Supplementary Figure 2a.pdf]
