# Supplementary material for: Structural basis of QueC-family protein function in qatABCD anti-phage defense
Source: Nat Commun. 2026 Apr 20;17:5420. doi: 10.1038/s41467-026-72155-8 (PMC13279940; doi:10.1038/s41467-026-72155-8)
Supplement: Supplementary file 6 — Transparent Peer Review file [file 41467_2026_72155_MOESM6_ESM.pdf]

nature portfolio

Peer Review File

Structural basis of QueC-family protein function in qatABCD anti-phage defense

Corresponding Author: Professor Philip Kranzusch

This file contains all reviewer reports in order by version, followed by all author rebuttals in order by version.

Version 0:

Reviewer comments:

Reviewer #1

(Remarks to the Author)
The study investigates the qatABCD system, focusing on the role of QatC (a QueC-family protein) and its interaction with QatB (an immunity protein). The authors show that QatC forms a specific complex with QatB, confirming their physical and functional association within the qatABCD defense system. QatBC complex is the minimal functional unit required for the antiviral defense mechanism. QatC is a QueC homolog that operates analogously within the qatABCD system as it does in CBASS, through deazaguanylation.Following the recent discovery of deazaguanylation, the results highlight a more general role for QueC-like enzymes in diverse bacterial immune systems.

This was a wonderful article to read. It is well-written and conceptually clear, with all major claims supported by well-designed experiments and high-quality figures. The structural and biochemical analysis of the QatBC complex provides deep insight into conserved domains and the catalytic mechanism of QueC-family proteins.
The article can be published under this form, we just noted very minor virus taxonomy details that could be updated. Congratulations to all the authors on this study.

Minor Comments and Suggestions
• Line 37: Replace “to synthesize to the key nucleobase” with “to synthesize the key nucleobase.”
• Line 70 / Figure 1: The viral families cited from Maffei, Shaidullina et al. (2021)—Siphoviridae, Myoviridae, and Autographiviridae—are outdated per the current ICTV virus taxonomy. Consider grouping phages by morphotype instead (e.g., Siphovirus, Myovirus, Podovirus).
• SECphi17 classification: This phage is not part of Caudoviricetes and therefore maybe should not be grouped with other Podoviruses.

Reviewer #2

(Remarks to the Author)
I co-reviewed this manuscript with one of the reviewers who provided the listed reports. This is part of the Nature Communications initiative to facilitate training in peer review and to provide appropriate recognition for Early Career Researchers who co-review manuscripts.

Reviewer #3

(Remarks to the Author)
This manuscript by Gao et al. represents a notable advance in our understanding of bacterial anti-phage defense systems. The study provides a detailed structural and functional characterization of the qatABCD system, identifying the QatB–QatC complex as its core and minimally sufficient functional unit. By drawing insightful parallels with the recently characterized type IV CBASS system, the authors convincingly demonstrate that QueC-family enzymes—traditionally associated with nucleoside biosynthesis—have been broadly repurposed in bacterial immunity to catalyze protein–nucleobase conjugation reactions. The structural data are of high quality, the experiments are carefully designed, and the conclusions are well supported by the evidence. It establishes a new functional class within bacterial defense systems (QueC-dependent protein modifiers) and provides a clear structural and mechanistic framework for future studies on qatABCD and related systems.

Suggestions for consideration:

1)The mechanism by which the proposed modification of QatB results in phage restriction remains somewhat unclear. Moreover, the functions of the putative “effector” proteins QatA (NTPase) and QatD (nuclease) are still enigmatic, as their deletion did not abolish defense in the reported assays. It would be helpful if the authors could comment on whether these proteins might serve auxiliary roles, contribute to defense against other phages, or require specific activation conditions.

2)In Fig. S1, the qatA mutant appears to display a partial loss of antiphage activity, yet the deletion of qatA was reported to have no significant effect. Could the authors clarify this apparent discrepancy and discuss whether QatA and QatD might contribute to defense under different phage challenges?

Reviewer #4

(Remarks to the Author)
This manuscript presents a detailed structural and functional investigation of the qatABCD anti-phage defense system, with a focus on the QatBC complex. The study provides structural data and identifies QatB, a previously uncharacterized protein bearing a conserved MGTS motif, as a likely substrate and binding partner of the QueC-family enzyme QatC. This work contributes to the growing understanding of how QueC-like proteins function beyond nucleoside biosynthesis, highlighting their role in post-translational modification within bacterial immune systems. The demonstration that QatBC alone is sufficient for anti-phage defense is a key finding. However, the mechanistic insights remain somewhat limited, as the exact nature of the chemical modification catalyzed by QatC is not resolved, and the broader functional consequences on phage restriction are not fully explored. In addition, the quality of crystallographic data is not sufficient and the models require further refinement. The comments below aim to address these points and suggest revisions to enhance the clarity, rigor, and overall impact of the work.

1. Please specify the source organism of the QatB and QatC proteins in the abstract to provide appropriate biological context
2. Line 22: The term 'immunity protein QatB' in the abstract is too general; please consider specifying its functional role more precisely.
3. Lines 38–39: The manuscript describes the two-step, ATP-dependent reaction catalyzed by QueC, converting CDG to $preQ_0$ via an ADG intermediate. However, this complex mechanism is not illustrated. Could the authors include a schematic figure showing the catalytic steps of QueC, including the conversion of CDG → ADG → $preQ_0$, role of both ATP molecules, and the incorporation of ammonia. Such a figure would enhance clarity for readers and provide a useful biochemical context for comparing canonical QueC with modified functions in Cap9 and QatC.
4. Lines 43–45, 55–57: The manuscript draws parallels between Cap9 and QatC-mediated modification. Is there direct biochemical evidence (e.g., mass spectrometry or chemical labeling) that confirms QatC modifies QatB via a covalent deazaguanine conjugation, analogous to NDG?
5. Line 56: Since QatC is proposed as a functional analog of Cap9, it would be important to report the sequence identity between the two proteins, and this should be mentioned in the text.
6. Lines 76-77: The sentence stating that "QatA is capable of multimerization and can bind both single-stranded and double-stranded DNA...” does not appear to contribute meaningfully to the central findings of the manuscript, which focuses on the QatBC proteins. Since QatA is not the primary subject of this study, and the described activities are not explored further or linked to the core conclusions, this data feels out of place. I recommend removing this information unless a clearer functional or mechanistic relevance to QatBC can be established.
7. Line 80-83: this can be shortened to say that A and D deletions in both Pseudomonas and E. coli preserve protective functions.
8. Line 81: While QatBC are sufficient, is there any evidence for either of the proteins being essential?
9. Lines 93–94: The crystal structure reveals that QatB and QatC form a 1:1 complex; however, it is unclear whether this stoichiometry is maintained under physiological conditions. Have the authors confirmed that the QatBC complex exists as a 1:1 assembly in solution using SEC-MALS, or native mass spectrometry? Providing such data would strengthen the physiological relevance of the crystallographic findings. Also, please clarify what is the oligomeric state of QatB and QatC proteins?
10. Line 100-102: A figure clearly showing the Zn^{2+} coordination by the conserved QatC cysteines (C334, C354, C357, and C360) would enhance the reader’s understanding and should be included.
11. Lines 100-102: The manuscript mentions four conserved cysteines (C334, C354, C357, and C360) forming a Zn^{2+}-binding site in QatC; however, this is not immediately clear from the sequence alignment provided (supplementary fig. 3). To improve clarity for readers, the authors should specifically highlight or annotate these cysteine residues in the sequence alignment figure, making it easier to locate. In Supplementary Figures 2 and 3, the critical residues described in the main text should be clearly annotated and visually enhanced, so they are easily identifiable and stand out.
12. Line 108-110: The current description of interactions between QatC and QatB, focusing on charged surface patches, lacks sufficient detail regarding the specific types of molecular interactions. Could the authors provide a more comprehensive analysis, including potential hydrogen bonds (e.g., between QatB N175 and QatC R414, and between QatC H413 and QatB D181) as well as possible water-mediated contacts observed at the interface?
13. Lines 110-113: The manuscript states that the QatC loop (residues N265–H282) inserts into a hydrophobic pocket of QatB, but this interaction is not described in sufficient detail. Please provide a description of the hydrophobic pocket, including the specific QatB residues involved. Additionally, QatB residues Q171 and Q234 are reported to form hydrogen bonds—please clarify which QatC residues these interact with. Notably, Q171 and Q234 of QatB are not labeled in Figure 2d; the authors should label these residues as well as the QatC loop residues to improve clarity in the figure.

14. Lines 122-124: The electron density map around the phosphate groups of the ATP ligand shows negative difference density, indicating possible inaccuracies in the current ligand fitting. I recommend further refinement and careful modeling of the ATP molecule to ensure an accurate representation of its conformation in the active site. Perhaps the occupancy is less than 1? Partial hydrolysis? Please, include a figure with an omit map for the ligand.
15. Lines 137-139: The manuscript mentions that Tyr and Asp residues coordinate the NDG nucleobase in the Cap9 structure; however, this should be described more precisely. Specifically, does Y115 form a π–π stacking interaction with the NDG nucleobase, and does D117 form a hydrogen bond with a nitrogen atom on its Watson–Crick edge? These interactions should be explicitly described in the text.
16. The manuscript refers to 'Tyr and Asp' residues involved in NDG coordination in the Cap9 structure, but the specific residue numbers (Y115 and D117) should be stated.
17. Lines 141-142: Have the authors considered other residues within the substrate-binding pocket that might interact with CDG, such as the tyrosine residues at positions 284 and 285, which are located near the threonines in QatC? Could these tyrosines potentially contribute to substrate binding or recognition in QatC.
18. Lines 141-143: The substitution of conserved residues with two threonine's (T278 and T280) in QatC represents an important difference. To highlight this key divergence, the authors should annotate these residues in the sequence alignment figure, ensuring they immediately stand out to readers.
19. lines 171-175: The authors report that the AlphaFold 3 model places the truncated QatB N-terminal residues inside the QatC active site; however, given that the N-terminal 65 residues of QatB exhibit low confidence scores in the model, how reliable is this predicted interaction? Could the authors discuss the limitations of the model in this region.
20. Lines 182-185: The manuscript describes deletion mutants of QatB targeting residues 16–22 and 34–40, with functional consequences observed only for the deletion of the conserved 16–22 segment. However, the rationale for selecting these specific regions for deletion is not clearly explained.
21. In Fig. 1b, expression of qatABCD does not reduce plaque number or size for phages Bas17 and Bas21 (Siphoviridae family), at least by my judgement, yet this observation is not addressed in the main text. Is there a better way to quantify this type of data to avoid subjective estimates?
22. In Figure 2a and d, labeling the secondary structure elements would help relate the figure more clearly to the structural description provided in the text.
23. Figure 2b would benefit from a structural superposition of QatC with QueC and Cap9 to enable direct comparison of their structural similarities and differences. Including RMSD values from these alignments would further support the structural analysis.
24. In Figure 2d, hydrogen bonds should be shown as dotted lines to clearly illustrate the specific interactions at the QatB–QatC interface. Additionally, the loop region residues of QatC should be labeled, and residues Q171 and Q234 of QatB, mentioned in the main text should be indicated in the figure to support the structural interpretation.
25. There are several concerns regarding the crystallographic data and refinement statistics reported in Supplemental Table S2 for the QatB–QatC structures:
Resolution cutoffs and data quality in the highest resolution shell:
For the apo structure, the CC1/2 value in the highest resolution shell is 0.303, and completeness is only 29%, which strongly suggests that this shell should be excluded from refinement. Although the R-free value for the highest shell is reported as 0.0910, which seems unusually low, only 3 reflections were used for R-free calculations in that shell. This extremely small number likely renders the R-free value statistically unreliable, typically at least 100 reflections are needed. For the ATP-bound structure, the CC1/2 in the highest shell is 0.368, again below the standard 0.5 cutoff. Besides CC1/2 other parameters also indicate that the claimed resolution is a stretch (low mean I/sigmaI).
Refinement and model quality: I find R factors, especially for the higher resolution structure, unsatisfactory. I would strongly recommend revisiting the revisiting the model fit to the map and crystallographic refinement strategy. Looking at the maps, possibly more residues could be modeled in the missing loops, some side chains appear to have double conformations, not currently accounted for, main chain of section R271-R277 possibly also in double confirmation. I suspect Pro372 to be in cis configuration (modeled as trans), and there are other instances where fit to the map can be improved. Regarding the crystallographic refinement – the authors don't provide the detailed calculation strategy in the Methods. From the PDB header it seems TLS refinement was used. For 1.3 A structure full anisotropic refinement should be done. I don't know where the final resolution cut off will be, but I expect it to be in the 1.4 – 1.5 A range, which will also allow for full anisotropic refinement, or at least for protein atoms only if the calculation doesn't converge.
Formatting and consistency issues: The space group should be formatted as $P2_12_12_1$, not "P 21 21 21". Wavelength (unit?) and other numbers should be given with consistent decimal precision (more is not necessarily better). Units should be included for RMSD (bonds) (Å), RMSD (angles) (degrees), B-factors ($Å^2$), and unit cell lengths (Å). The nucleic acid row can be removed since no nucleic acids are present. Unit cell angles are listed for both structures, however 90deg is expected for orthorhombic space groups so it could be omitted.
Minor comments
1. For clarity, please color the zinc ion in the figures using a more contrasting color that clearly differentiates it from surrounding residues and ligands.
2. In line 37, the phrase 'required to synthesize to the key nucleobase' should be revised by removing 'to'
3. Line 99: To be more precise, I'd say "composed of a five-stranded, parallel β-sheet (β5-β9)"
4. Duplicated word "that" in line 211
5. In line 386, formatting should be corrected: '100x15mm' should be written as '100x15 mm', and similarly, '120x120mm' should be formatted as '120x120 mm'
6. In Table 1, the organism names should be formatted in italics, following standard taxonomic conventions.

Reviewer #5

(Remarks to the Author)
I co-reviewed this manuscript with one of the reviewers who provided the listed reports. This is part of the Nature Communications initiative to facilitate training in peer review and to provide appropriate recognition for Early Career Researchers who co-review manuscripts.

Version 1:

Reviewer comments:

Reviewer #1

(Remarks to the Author)
The manuscript was already publishable almost as is for us before reviews.
No additionnal commments.

Reviewer #2

(Remarks to the Author)
I co-reviewed this manuscript with one of the reviewers who provided the listed reports. This is part of the Nature Communications initiative to facilitate training in peer review and to provide appropriate recognition for Early Career Researchers who co-review manuscripts.

Reviewer #3

(Remarks to the Author)
The authors have satisfactorily addressed all of my concerns.

Reviewer #4

(Remarks to the Author)
I am satisfied with the authors' corrections to the manuscript and appreciate the time and effort invested in conducting additional experiments and improving the structure. I fully acknowledge the limitations of some of the approaches taken. One general comment I would like to make is that the PDB now offers deposit versioning (https://www.rcsb.org/docs/exploring-a-3d-structure/versions), which might have been a more suitable option for uploading the updated structural information rather than creating entirely new deposits. However, I understand that it may not be feasible to rework this at this stage.

Reviewer #5

(Remarks to the Author)
I co-reviewed this manuscript with one of the reviewers who provided the listed reports. This is part of the Nature Communications initiative to facilitate training in peer review and to provide appropriate recognition for Early Career Researchers who co-review manuscripts.

Open Access This Peer Review File is licensed under a Creative Commons Attribution 4.0 International License, which permits use, sharing, adaptation, distribution and reproduction in any medium or format, as long as you give appropriate credit to the original author(s) and the source, provide a link to the Creative Commons license, and indicate if changes were made.
In cases where reviewers are anonymous, credit should be given to 'Anonymous Referee' and the source.
The images or other third party material in this Peer Review File are included in the article's Creative Commons license, unless indicated otherwise in a credit line to the material. If material is not included in the article's Creative Commons license and your intended use is not permitted by statutory regulation or exceeds the permitted use, you will need to obtain permission directly from the copyright holder.
To view a copy of this license, visit https://creativecommons.org/licenses/by/4.0/

Reviewer #1:
The study investigates the qatABCD system, focusing on the role of QatC (a QueC-family protein) and its interaction with QatB (an immunity protein). The authors show that QatC forms a specific complex with QatB, confirming their physical and functional association within the qatABCD defense system. QatBC complex is the minimal functional unit required for the antiviral defense mechanism. QatC is a QueC homolog that operates analogously within the qatABCD system as it does in CBASS, through deazaguanylation.Following the recent discovery of deazaguanylation, the results highlight a more general role for QueC-like enzymes in diverse bacterial immune systems.

This was a wonderful article to read. It is well-written and conceptually clear, with all major claims supported by well-designed experiments and high-quality figures. The structural and biochemical analysis of the QatBC complex provides deep insight into conserved domains and the catalytic mechanism of QueC-family proteins. The article can be published under this form, we just noted very minor virus taxonomy details that could be updated.

Congratulations to all the authors on this study.

We thank the reviewer for their positive evaluation of our manuscript, and we are grateful for their helpful feedback.

Minor Comments and Suggestions
• Line 37: Replace "to synthesize to the key nucleobase" with "to synthesize the key nucleobase."

We thank the reviewer and have corrected the text as suggested.

• Line 70 / Figure 1: The viral families cited from Maffei, Shaidullina et al. (2021)—Siphoviridae, Myoviridae, and Autographiviridae—are outdated per the current ICTV virus taxonomy. Consider grouping phages by morphotype instead (e.g., Siphovirus, Myovirus, Podovirus).

We thank the reviewer for pointing out this error. We have updated the manuscript text to refer to phages by morphotype (see Lines 69–70) and adjusted the organization of phages in Fig. 1b accordingly. We have also clarified that phages are grouped by morphology in the figure captions (see Lines 347–349, 568-570).

• SECphi17 classification: This phage is not part of Caudoviricetes and therefore maybe should not be grouped with other Podoviruses.

We have updated Fig. 1b to separate phage SECphi17 from the dsDNA phages.

Reviewer #2:
I co-reviewed this manuscript with one of the reviewers who provided the listed reports.

We thank the reviewer for their help reviewing our manuscript.

Reviewer #3:
This manuscript by Gao et al. represents a notable advance in our understanding of bacterial anti-phage defense systems. The study provides a detailed structural and functional characterization of the qatABCD system, identifying the QatB–QatC complex as its core and minimally sufficient functional unit. By drawing insightful parallels with the recently characterized type IV CBASS system, the authors convincingly demonstrate that QueC-family enzymes—traditionally associated with nucleoside biosynthesis—have been broadly repurposed in bacterial immunity to catalyze protein–nucleobase conjugation reactions. The structural data are of high quality, the experiments are carefully designed, and the conclusions are well supported by the evidence. It establishes a new functional class within bacterial defense systems (QueC-dependent protein modifiers) and provides a clear structural and mechanistic framework for future studies on qatABCD and related systems.

We thank the reviewer for highlighting our study as a notable advance in anti-phage defense systems, and we are grateful for their helpful suggestions to improve the manuscript.

Suggestions for consideration:
1) The mechanism by which the proposed modification of QatB results in phage restriction remains somewhat unclear. Moreover, the functions of the putative "effector" proteins QatA (NTPase) and QatD (nuclease) are still enigmatic, as their deletion did not abolish defense in the reported assays. It would be helpful if the authors could comment on whether these proteins might serve auxiliary roles, contribute to defense against other phages, or require specific activation conditions.

We agree with the reviewer that the mechanism by which qatABCD restricts phage infection is of great interest. Due to the uncertainty of the nature of the QatB modification, we have avoided speculating on how this modification may result in phage restriction. We also note that in type IV CBASS, where the N-terminal modification has been identified, the mechanism by which the protein modification activates anti-phage defense remains unknown. This activation mechanism will be a major focus of future work.

To address the reviewer's comments on the functions of QatA and QatD, we have performed a new experiment where we assess the anti-phage defense activity of the full *P. aeruginosa* qatABCD operon, as well as *P. aeruginosa* and *E. coli* operons in which QatA and QatD have been deleted, against our full panel of 13 phages (Supplementary Fig. 3). We observed that *P. aeruginosa* QatB and QatC alone retain strong defense against some phages such as λ_{vir} and $P1_{vir}$, but exhibit reduced or no defense against others such as Bas25 and Bas39. These results suggest that QatB and QatC are sufficient to defend against some, but not all, phages, and that QatA and QatD may expand the range of viruses which the qatABCD operon can defend against. We have noted these new observations in Lines 83–86.

2) In Fig. S1, the qatA mutant appears to display a partial loss of antiphage activity, yet the deletion of qatA was reported to have no significant effect. Could the authors clarify this apparent discrepancy and discuss whether QatA and QatD might contribute to defense under different phage challenges?

We agree with the reviewer that the QatA mutant displays a partial loss of antiphage activity. We apologize if our original wording implied that deletion of QatA alone did not have any significant effect on defense. We have adjusted the wording in Lines 77–78 and 243–245 to clarify this point and have updated our discussion on the role of QatA and QatD as described in the response to reviewer point #1.

Reviewer #4:

This manuscript presents a detailed structural and functional investigation of the qatABCD anti-phage defense system, with a focus on the QatBC complex. The study provides structural data and identifies QatB, a previously uncharacterized protein bearing a conserved MGTS motif, as a likely substrate and binding partner of the QueC-family enzyme QatC. This work contributes to the growing understanding of how QueC-like proteins function beyond nucleoside biosynthesis, highlighting their role in post-translational modification within bacterial immune systems. The demonstration that QatBC alone is sufficient for anti-phage defense is a key finding. However, the mechanistic insights remain somewhat limited, as the exact nature of the chemical modification catalyzed by QatC is not resolved, and the broader functional consequences on phage restriction are not fully explored. In addition, the quality of crystallographic data is not sufficient and the models require further refinement. The comments below aim to address these points and suggest revisions to enhance the clarity, rigor, and overall impact of the work.

We thank their reviewer for their helpful comments and for highlighting the sufficiency of QatBC for anti-phage defense as a key finding of our manuscript.

1. Please specify the source organism of the QatB and QatC proteins in the abstract to provide appropriate biological context

We have made the requested change in Line 21-22.

2. Line 22: The term 'immunity protein QatB' in the abstract is too general; please consider specifying its functional role more precisely.

We agree with the reviewer that the term "immunity protein" is a general term. Prior work has not been able to identify a functional role for QatB, and QatB is best known to the field as a protein of unknown function that is associated with phage defense. Therefore, we decided to refer to QatB as an immunity protein as this description best reflects existing knowledge in the field.

3. Lines 38–39: The manuscript describes the two-step, ATP-dependent reaction catalyzed by QueC, converting CDG to $preQ_0$ via an ADG intermediate. However, this complex mechanism is not illustrated. Could the authors include a schematic figure showing the catalytic steps of QueC, including the conversion of CDG → ADG → $preQ_0$, role of both ATP molecules, and the incorporation of ammonia. Such a figure would enhance clarity for readers and provide a useful biochemical context for comparing canonical QueC with modified functions in Cap9 and QatC.

We thank the reviewer for their thoughtful suggestion and have included an additional supplementary figure depicting the catalytic steps of QueC (see Supplementary Fig. 1).

4. Lines 43–45, 55–57: The manuscript draws parallels between Cap9 and QatC-mediated modification. Is there direct

biochemical evidence (e.g., mass spectrometry or chemical labeling) that confirms QatC modifies QatB via a covalent deazaguanine conjugation, analogous to NDG?

We agree with the reviewer that direct detection of QatB modification is of key interest, but we have not been able to identify conditions that promote modification.

One possibility is that QatC utilizes a substrate other than CDG, as noted in Lines 154–155. To illustrate this, we have performed a new experiment where we mutate the conserved T278 and T280 residues in the QatC active site (Figure 3c). In canonical QueC enzymes and in Cap9, these positions are occupied by conserved Tyr and Asp residues that mediate recognition of CDG. We observed that T278A/T280A and T278Y/T280D mutations both result in loss of phage defense, suggesting that these residues are necessary for potential substrate recognition. However the identity of this substrate and the resulting protein modification remain unknown at this time.

5. Line 56: Since QatC is proposed as a functional analog of Cap9, it would be important to report the sequence identity between the two proteins, and this should be mentioned in the text.

We thank the reviewer for this suggestion and have made the requested change in Line 105.

6. Lines 76-77: The sentence stating that "QatA is capable of multimerization and can bind both single-stranded and double-stranded DNA..." does not appear to contribute meaningfully to the central findings of the manuscript, which focuses on the QatBC proteins. Since QatA is not the primary subject of this study, and the described activities are not explored further or linked to the core conclusions, this data feels out of place. I recommend removing this information unless a clearer functional or mechanistic relevance to QatBC can be established.

We agree with the reviewer that QatA is discussed in limited detail in the manuscript. However, we believe these novel findings provide important context to our discovery that QatA is not uniformly required for phage defense. Previous and current work on qatABCD (Gao et al. *Science* 2020 PMID 32855333; Oh and Bae *bioRxiv* 2025 doi: 10.1101/2025.09.22.677948; Gao et al *bioRxiv* 2025 doi: 10.1101/2025.11.03.686418) presumed that QatA was an essential effector, so inclusion of this data will help guide future experiments in the field. To improve clarity, we have removed the *qatA*$^{\Delta 501–649}$ mutant as it is not discussed in the main text (Supplementary Fig. 1c).

7. Line 80-83: this can be shortened to say that A and D deletions in both Pseudomonas and E. coli preserve protective functions.

We agree that it is possible to condense this segment, but we believe that the observation that QatB and QatC alone are capable of mediating defense against phage λ_{vir} is a key finding of this manuscript and prefer to retain our emphasis on this point.

8. Line 81: While QatBC are sufficient, is there any evidence for either of the proteins being essential?

Yes, QatB and QatC are both essential for phage defense. We show that deletion of QatB results in loss of phage defense. We also show that truncation to a conserved region of the QatB tail or a point mutation to the conserved G2 glycine similarly result in loss of phage defense (Fig. 4d). This indicates that QatB is essential and highlights the key required features of QatB. Similarly, we show that mutation of key residues in the QatC active site results in loss of phage defense, indicating that QatC activity is essential for qatABCD function (Fig. 3c). We have adjusted our wording on Lines 157-158 and 193-194 to improve clarity on this point.

9. Lines 93–94: The crystal structure reveals that QatB and QatC form a 1:1 complex; however, it is unclear whether this stoichiometry is maintained under physiological conditions. Have the authors confirmed that the QatBC complex exists as a 1:1 assembly in solution using SEC-MALS, or native mass spectrometry? Providing such data would strengthen the physiological relevance of the crystallographic findings. Also, please clarify what is the oligomeric state of QatB and QatC proteins?

To address this reviewer's point, we have performed mass photometry on the QatBC complex and confirmed that it is a 1:1 assembly (Supplementary Fig. 4). We have additionally performed mass photometry on QatA and observed that this protein forms a heptamer or octamer in solution (Supplementary Fig. 2b).

10. Line 100-102: A figure clearly showing the Zn^{2+} coordination by the conserved QatC cysteines (C334, C354, C357, and C360) would enhance the reader's understanding and should be included.

We thank the reviewer for their suggestion. We have added a new panel showing Zn^{2+} coordination (Supplementary Fig. 7a).

11. Lines 100-102: The manuscript mentions four conserved cysteines (C334, C354, C357, and C360) forming a Zn^{2+}-binding site in QatC; however, this is not immediately clear from the sequence alignment provided (supplementary fig. 3). To improve clarity for readers, the authors should specifically highlight or annotate these cysteine residues in the sequence alignment figure, making it easier to locate. In Supplementary Figures 2 and 3, the critical residues described in the main text should be clearly annotated and visually enhanced, so they are easily identifiable and stand out.

We thank the reviewer for their helpful suggestion. We have highlighted the QatB residues which mediate interaction with QatC (Supplementary Fig. 5). In addition, we have highlighted the QatC residues which are involved in Zn^{2+} binding, ATP binding, and interaction with QatB. We have also highlighted the T278 and T280 residues which align with the CDG-binding residues in QueC and Cap9 (Supplementary Fig. 6). Finally, we re-numbered both alignments to match the residue numbers of the *P. aeruginosa* QatB and QatC sequences to improve clarity.

12. Line 108-110: The current description of interactions between QatC and QatB, focusing on charged surface patches, lacks sufficient detail regarding the specific types of molecular interactions. Could the authors provide a more comprehensive analysis, including potential hydrogen bonds (e.g., between QatB N175 and QatC R414, and between QatC H413 and QatB D181) as well as possible water-mediated contacts observed at the interface?

We have updated the text to describe specific interactions between residue side chains in these region (see Lines 115–116). These interactions do not appear to be water-mediated.

13. Lines 110-113: The manuscript states that the QatC loop (residues N265–H282) inserts into a hydrophobic pocket of QatB, but this interaction is not described in sufficient detail. Please provide a description of the hydrophobic pocket, including the specific QatB residues involved. Additionally, QatB residues Q171 and Q234 are reported to form hydrogen bonds—please clarify which QatC residues these interact with. Notably, Q171 and Q234 of QatB are not labeled in Figure 2d; the authors should label these residues as well as the QatC loop residues to improve clarity in the figure.

We have updated the text in Lines 116–120 to describe the interaction between the QatC loop and QatB in more detail and updated Fig. 2d to label the sidechains of the described residues. Although additional hydrophobic packing and hydrogen-bond interactions occur along this interface, we have elected to focus on this subset to maintain clarity of the figure and text.

14. Lines 122-124: The electron density map around the phosphate groups of the ATP ligand shows negative difference density, indicating possible inaccuracies in the current ligand fitting. I recommend further refinement and careful modeling of the ATP molecule to ensure an accurate representation of its conformation in the active site. Perhaps the occupancy is less than 1? Partial hydrolysis? Please, include a figure with an omit map for the ligand.

We have added a figure showing an omit map of ATP and Mg^{2+} (Supplemental Fig. 7b).

15. Lines 137-139: The manuscript mentions that Tyr and Asp residues coordinate the NDG nucleobase in the Cap9 structure; however, this should be described more precisely. Specifically, does Y115 form a π–π stacking interaction with the NDG nucleobase, and does D117 form a hydrogen bond with a nitrogen atom on its Watson–Crick edge? These interactions should be explicitly described in the text.

We have adjusted the text in Lines 146–149 to clarify how these two Cap9 residues bind with the NDG nucleobase.

16. The manuscript refers to 'Tyr and Asp' residues involved in NDG coordination in the Cap9 structure, but the specific residue numbers (Y115 and D117) should be stated.

We thank the reviewer for their suggestion to clarify the manuscript, and we have made the requested change in Lines 147-150.

17. Lines 141-142: Have the authors considered other residues within the substrate-binding pocket that might interact with CDG, such as the tyrosine residues at positions 284 and 285, which are located near the threonines in QatC? Could these tyrosines potentially contribute to substrate binding or recognition in QatC.

In the currently observed QatC conformation, residues Y284 and Y285 are distant from the substrate binding pocket and blocked by the loop containing residues T278 and T280. As also described in our response to reviewer's point #4, we have included new data showing that mutation of these threonine residues eliminates defense, implicating them in substrate recognition and QatC activity. However, we cannot rule out at this time that a conformational change may allow residues Y284 and Y285 to be involved in substrate binding.

18. Lines 141-143: The substitution of conserved residues with two threonine's (T278 and T280) in QatC represents an important difference. To highlight this key divergence, the authors should annotate these residues in the sequence alignment figure, ensuring they immediately stand out to readers.

We thank the reviewer for their helpful suggestion. As also described in response to reviewer's point #11, we have now highlighted T278 and T280 in the QatC alignment.

19. lines 171-175: The authors report that the AlphaFold 3 model places the truncated QatB N-terminal residues inside the QatC active site; however, given that the N-terminal 65 residues of QatB exhibit low confidence scores in the model, how reliable is this predicted interaction? Could the authors discuss the limitations of the model in this region.

We thank the reviewer for their feedback on this point. Given that the N-terminal 65 residues of QatB form a flexible tail it is not surprising that regions of the tail exhibit low confidence scores. However, the regions of the tail that are predicted to interface with QatC are predicted with high confidence. We have added a sentence in the main text to address this (see Lines 188–190). Recent QatBC structures from other groups released on *bioRxiv* shortly after our preprint was posted also support the placement of the N-terminal tail in the QatB active site (Oh and Bae bioRxiv 2025 doi: 10.1101/2025.09.22.677948; Gao et al bioRxiv 2025 doi: 10.1101/2025.11.03.686418). We have added a sentence in the discussion that references these corroborating studies (see Lines 229–231).

20. Lines 182-185: The manuscript describes deletion mutants of QatB targeting residues 16–22 and 34–40, with functional consequences observed only for the deletion of the conserved 16–22 segment. However, the rationale for selecting these specific regions for deletion is not clearly explained.

We thank the reviewer for pointing out our lack of clarity on this point. We deleted residues 16–22 in order to determine whether conserved residues on the N-terminal tail in addition to the MGTS motif were important for QatB function. We deleted the variable region 34–40 in order to demonstrate that the loss of defense upon deletion of residues 16–22 is not simply a result of a change in tail length. We have adjusted our wording in Lines 199–202 to clarify.

21. In Fig. 1b, expression of qatABCD does not reduce plaque number or size for phages Bas17 and Bas21 (Siphoviridae family), at least by my judgement, yet this observation is not addressed in the main text. Is there a better way to quantify this type of data to avoid subjective estimates?

We agree with the reviewer that expression of *P. aeruginosa* qatABCD operon does not reduce plaque number or size for phages Bas17 and Bas21. These phages are included to show that qatABCD does not defend against all phages. Because siphovirus is a morphological classification and not a phylogenetic one, it is not surprising that qatABCD only defends against a subset of the tested siphoviruses. To clarify this, we have corrected the outdated *Siphoviridae* and *Myoviridae* classifications and instead referred to the viruses by morphotypes (see Lines 69–70). We have also clarified that viruses are grouped by morphology in the figure caption (see Lines 348 and 569).

We also agree with the reviewer that analysis of the plaque assay data is subjective. The standard method of quantification is to compare phage plaque forming units (PFU). However, this method is inherently subjective as many experiments to do not produce countable plaques (see *P. aeruginosa* qatABCD defense against Bas25 in Supplemental Fig. 3), and it does not take into account decreases in plaque size that result from defense. For these reasons, we prefer to display the original plaque assay data, which allows the reader to directly interpret defense from the primary data.

22. In Figure 2a and d, labeling the secondary structure elements would help relate the figure more clearly to the structural description provided in the text.

Secondary structure is shown with protein sequence in Supplemental Figures 5 and 6. We have added relevant secondary structure labels in Fig. 2d and Fig. 2e. Due to the large number of secondary structure elements in Fig. 2a we prefer to leave out these labels to prevent obscuring the displayed structure.

23. Figure 2b would benefit from a structural superposition of QatC with QueC and Cap9 to enable direct comparison of their structural similarities and differences. Including RMSD values from these alignments would further support the structural analysis.

We thank the reviewer for their suggestion. We have included a superposition of QatC, QueC, and Cap9 and displayed the RMSD values (Fig. 2b).

24. In Figure 2d, hydrogen bonds should be shown as dotted lines to clearly illustrate the specific interactions at the QatB–

QatC interface. Additionally, the loop region residues of QatC should be labeled, and residues Q171 and Q234 of QatB, mentioned in the main text should be indicated in the figure to support the structural interpretation.

As discussed above in response to reviewer point #13, we have updated this figure with labels for the specific interactions that are referenced in the text. Due to the large number of residues involved in this loop and the protein-protein interface, it is not feasible to fully display and label every residue without obscuring the structure. We have chosen this hybrid approach of including some labels to allow the reader to see the important interactions in the context of the full interface.

25. There are several concerns regarding the crystallographic data and refinement statistics reported in Supplemental Table S2 for the QatB–QatC structures:

Resolution cutoffs and data quality in the highest resolution shell:
For the apo structure, the CC1/2 value in the highest resolution shell is 0.303, and completeness is only 29%, which strongly suggests that this shell should be excluded from refinement. Although the R-free value for the highest shell is reported as 0.0910, which seems unusually low, only 3 reflections were used for R-free calculations in that shell. This extremely small number likely renders the R-free value statistically unreliable, typically at least 100 reflections are needed. For the ATP-bound structure, the CC1/2 in the highest shell is 0.368, again below the standard 0.5 cutoff. Besides CC1/2 other parameters also indicate that the claimed resolution is a stretch (low mean I/sigmaI).

We thank the reviewer for their helpful feedback to improve our structural analyses. The reflection data for the apo structure was originally processed using STARANISO as part of the autoPROC pipeline (Vonrhein 2011 PMID 21460447; Global Phasing Limited), and the anisotropic high resolution was selected based on the default criteria of $CC_{1/2} \geq 0.3$ and local I/sigmaI ≥ 1.2 (Karplus 2016 PMID 26209821). The low number of R-free reflections originally reported in the high resolution shell was an error due to incorrect labeling of the resolution shells when generating the output table from Phenix and the true number of R-free reflections in the highest shell was 185 out of 3444 unique reflections with R-work / R-free of 0.299 / 0.315. We are grateful to the reviewer for their close evaluation which has allowed us to correct this mistake.

We also agree with the reviewer that the statistical strength of our models could be improved by reprocessing the original data with more conservative criteria for both the apo and ATP-bound structures. Based on their suggestions for $CC_{1/2}$ and I/sigmaI, we have reprocessed the data for both structures and set the high-resolution cutoff based on the following critera: isotropic resolution cutoff, $CC_{1/2} \geq 0.5$, I/sigmaI ≥1.5. The resulting resolutions were 1.55 Å for the apo structure and 1.81 Å for the ATP structure. The highest resolution shell of the apo structure now has a $CC_{1/2}$ of 0.767, completeness of 100%, and R-free of 0.2873 calculated from 182 reflections. The highest resolution shell for the ATP-bound structure now has a $CC_{1/2}$ of 0.778, completeness of 99.5%, and R-free of 0.2868 from 127 reflections (Supplementary Table 2). These structures have been submitted to PDB with new accession codes 9ZEE (ATP) and 9ZEF (apo).

Refinement and model quality: I find R factors, especially for the higher resolution structure, unsatisfactory. I would strongly recommend revisiting the revisiting the model fit to the map and crystallographic refinement strategy. Looking at the maps, possibly more residues could be modeled in the missing loops, some side chains appear to have double conformations, not currently accounted for, main chain of section R271-R277 possibly also in double confirmation. I suspect Pro372 to be in cis configuration (modeled as trans), and there are other instances where fit to the map can be improved. Regarding the crystallographic refinement – the authors don't provide the detailed calculation strategy in the Methods. From the PDB header it seems TLS refinement was used. For 1.3 A structure full anisotropic refinement should be done. I don't know where the final resolution cut off will be, but I expect it to be in the 1.4 – 1.5 A range, which will also allow for full anisotropic refinement, or at least for protein atoms only if the calculation doesn't converge.

We thank the reviewer for their suggestions. After reprocessing the apo structure, we were able to model in additional residues W182, D183 and S186 in QatC. We have also corrected P373 to the cis conformation in both structures. We were unable to improve the model in the 271–277 range by incorporating alternative conformations. It is likely that this region of the protein is somewhat flexible and might occupy several additional low occupancy states. We prefer to keep the currently modeled conformation as it is most strongly supported by the electron density. Similarly, for additional residues that may have alternative conformations, we prefer to model the major conformation most strongly supported by the electron density.

We thank the reviewer for their recommendations on refinement strategies. We have updated our Materials and Methods section to describe the selected refinement strategy in more detail (see Lines 467–473). At this resolution, we found that TLS parameters were sufficient to produce a high quality model and full anisotropic ADP refinement for all protein atoms yielded no significant improvement: R_{free} 0.1930 (0.2873) with TLS vs 0.1954 (0.2881) with all protein atom anisotropy for the apo structure.

Formatting and consistency issues: The space group should be formatted as $P2_12_12_1$, not "P 21 21 21". Wavelength (unit?) and other numbers should be given with consistent decimal precision (more is not necessarily better). Units should be included for RMSD (bonds) (Å), RMSD (angles) (degrees), B-factors (Å²), and unit cell lengths (Å). The nucleic acid row

can be removed since no nucleic acids are present. Unit cell angles are listed for both structures, however 90deg is expected for orthorhombic space groups so it could be omitted.

We thank the reviewer for their detailed feedback. We have made all of the requested corrections. Wavelengths are reported to 4 decimal points (Å) (Supplementary Table 2).

Minor comments
1. For clarity, please color the zinc ion in the figures using a more contrasting color that clearly differentiates it from surrounding residues and ligands.

We have updated all representations of the zinc ion to a contrasting light orange color.

2. In line 37, the phrase 'required to synthesize to the key nucleobase' should be revised by removing 'to'

We thank the reviewer for noting this error and we have made this correction.

3. Line 99: To be more precise, I'd say "composed of a five-stranded, parallel β-sheet (β5-β9)"

We have made this correction. We have also corrected similar text found on Line 111.

4. Duplicated word "that" in line 211

We thank the reviewer for their careful reading of the manuscript and we have made this correction.

5. In line 386, formatting should be corrected: '100x15mm' should be written as '100x15 mm', and similarly, '120x120mm' should be formatted as '120x120 mm'

We have made this change.

6. In Table 1, the organism names should be formatted in italics, following standard taxonomic conventions.

We thank the reviewer for pointing out this error. We have made the requested change.

Reviewer #5:
I co-reviewed this manuscript with one of the reviewers who provided the listed reports.

We thank the reviewer for their help reviewing our manuscript.
